# Within the Hospitals

**Published:** 1897-10-23

**Authors:** 


### The Institutional Workshop.

### WITHIN THE HOSPITALS.

### THE LONDON HOMŒOPATHIC HOSPITAL, GREAT ORMOND STREET.

In a notice which appeared in The Hospital for June 30th, 1894, of the proposed plan of this hospital, it is stated, "We shall look forward with much interest to seeing the completed plan." That pleasure we enjoyed a few days ago, when we made a thorough inspection of the new buildings from roof to basement. Our inspection confirms the opinion already expressed that great credit is due to Mr. William A. Pite, the architect, for the marked success with which he has accomplished a difficult task. On a narrow site, with all the hampering restrictions incident to a crowded district of London, Mr. Pite has erected a hospital building which contains wards which are as good as, or perhaps even better in some respects than, those of many recently-erected hospitals in this country. He has evinced marked skill in setting back the building so as to give light and air to the out-patient department, and in the methods of lighting that department from the rear which he has introduced. The building, now that it is completed, in view of the difficulties referred to, must constitute an object lesson for all who wish to study and understand modern hospital construction. We made a very few criticisms in dealing with the plan, but those criticisms have been reduced to insignificance by the changes the architect has since introduced. Mr. Pite has evidently made a study of the most recent hospital buildings, and anyone who wishes to benefit by the lessons which may be learnt from an inspection of the new Homœopathic Hospital, must of course be familiar with the principles of hospital construction in their present completeness. We have inspected hundreds of hospitals and kindred institutions, but we never remember before to have realised that there are lessons to be learnt in the board-room. The board-room at Great Ormond Street is perfectly proportioned, and it affords evidence of the loving and intelligent observation brought to bear by the officials, the committee, and the architect in settling the plans of the present building. The feature of the room is the fireplace, an old one, parts of which came from Arundel Castle. It is of a composite character, and has been built up from materials derived from various sources, which were obtained after diligent search, and this mantel and fireplace now constitute as a whole one of the most striking features in a really beautiful room. There has been an evident endeavour to provide that everything in the new building shall be of the best, and from the ingenious arrangement for the bottles containing the drugs in the dispensary to the mechanical contrivance in the gas stoves in the kitchen on the top floor, by which a large saving of gas is secured, this rare foresight must afford satisfaction to the trained observer as he goes from floor to floor.

#### A Comfortable Hospital.

The predominant impression conveyed to our minds by our inspection was that this is a comfortable hospital. Comfortable, that is to say, from the fact that everybody, as far as we can judge, who works within the institution is dominated by the feeling that upon his or her individual shoulders rests the responsibility to discharge their duties so as to secure the provision of all reasonable comfort for all, and the maximum of efficiency in every direction. Each ward contains provisions in the duty-rooms, and in the arrangements for the service of the dinners and other meals, which must add materially to the comfort of the patients by securing that the meals are served hot, and that they are presented to the patient in the form best calculated to be appetising and tempting. Then, again, the size and arrangements of the smaller wards containing nine beds are such that we incline to the feeling that wards of this size without screens for paying patients at, say, two guineas a week would be popular and satisfactory. The children's ward (an illustration of which we gave in The Hospital of June 19th, 1897) is one of the most attractive wards we have ever seen. A good friend of the hospital with artistic tendencies has designed and presented a large screen of plain glass which not only acts as a break at the entrance to the ward, but gives the whole room a special character which is striking and pleasant. Here, too, we find evidence of the thoughtful care which has been put into the arrangements for the comfort and well-being of the patients. Each child has its own clothes arranged separately and provided by the hospital. In those cases where the parents are too poor to adequately clothe the children, arrangements are made to allow each child to take out the suit used by it during the time of its residence in the hospital. These clothes are made from remnants obtained from certain firms who are interested in the hospital, and have a special character, which might be generally adopted by hospitals with advantage. Each garment is made of pieces, the pieces being of different patterns, but so selected as to make the general effect of the clothes unobtrusive and good. The object of this plan is to take it out of the power of the parents to pawn the clothes when the children leave the hospital. Pawnbrokers will not take clothes so constructed, and it is for this reason that we mention the matter here. Then, again, every child has its own towel, its own flannel, and its own brush and comb. The medicines are kept in a basket containing a separate division for each bed according to its number, and the poisons are safely locked up in a cupboard by themselves. The ward contains a portable cabinet with every requisite for tracheotomy operations, and the condition of the cots, the glass, and everything in the ward reflected great credit upon the nurses, who are deservedly proud of the spotless cleanliness, which is a great characteristic of this institution, and which is apparent when all due allowance has been made for the smartness which one expects to find in a new building. The same result was noticeable in the operation-room and in the condition of the instruments and the various vessels and appurtenances, and may be said to constitute a feature which is to be met with almost everywhere. The patients seemed to appreciate the comforts of their surroundings, which is not always the case.

### Some Anomalies.

Where there is so much to praise, as in a case like the building we are dealing with, it is remarkable to find certain defects which can easily be remedied, the presence of which is mainly due to the often unconscious effects of old customs or traditions. Thus, despite the fact that a change may represent a sum of £300 per annum, we were surprised to find that the committee of the Homœopathic Hospital still requires its patients to supply themselves with tea, sugar, and butter, a change of linen, with towels and soap, a brush and comb, and 1s. for breakages. It is due to the marked efficiency and intelligent thoughtfulness exhibited throughout the New Homœopathic Hospital that the authorities should make up their minds to abolish these requirements, and so place their hospital on a par with the most wisely administered hospitals in the country. Again, having so complete a children's ward, there is no excuse for permitting the adult patients in certain of the wards to be subject to the disturbing influence of suffering children, as is at present the case. Another objection to the presence of children in the adult wards is that this must necessarily consume some of the air allowance provided for the adults, and good wards of modern construction may be rendered unhealthy by a practice which tends to retard recovery, and is altogether indefensible where there is an adequate number of beds provided for children in a separate ward. Again, we are convinced that something may be done, despite the pressure of space, to remedy the untidiness of certain of the bathrooms, due to the presence of clothing and linen which are out of place there. Their presence may tend to render the hospital less hygienically complete than it otherwise would be. The communication between the mortuary and the hospital proper should be disconnected from the main building, as we understand it will be when the additions to the Nursing Home, now in course of construction, are completed.

### General Features.

We cannot justly conclude our notice of the Homœopathic Hospital without expressing our appreciation of the whole-hearted and intelligent services which the present secretary-superintendent, Mr. G. A. Cross, has rendered to the institution. Such of the books as we had occasion to examine, especially those relating to the admission of out-patients, and the register of attendance of the medical staff, are excellent of their kind, and were accurately and neatly kept. We observed that only about 2½ per cent. of the out-patients admitted are free cases, the larger proportion being persons who pay 1s. for a month's attendance, although a proportion are received by the presentation of governors' letters. The system on which this hospital is financed is notable too. There appears to be no attempt to appeal for an arbitrary sum, the amount of which seems often to be fixed for the reason that it is sufficient to excite sympathy and increase the amount of individual contributions. Mr. Cross and his committee wisely pursue the Spurgeon plan of setting forth clearly what money they require and the purposes to which that money will be devoted. In this way they have succeeded in paying off the whole of the building debt, and are at the present time steadily working to provide the funds for the erection of the additions to the Nursing Home, and to secure an income from annual subscriptions sufficient, with that derived from other sources, to maintain the hospital in efficiency. They appear to recognise, as men of business, that if you have an invested capital it is legitimate and proper to obtain the sanction of the governors to realise a sufficient amount of that capital from time to time to meet the necessary expenditure, and then to ask the public to enable them to replace the capital so expended and to maintain the reserve fund at an adequate amount.

Finally, we found that the whole of the drugs, apart from crude drugs, are given gratuitously to the hospital by a large firm of druggists, Messrs. E. Gould and Sons, 59, Moorgate Street, E.C., honorary chemists. We examined the drugs, and found them of excellent quality. Possibly other secretaries may like to know of this fact, as there must surely be some other firms who, if properly approached, would be willing to do as much for a hospital in which they may be interested as is done for the Homœopathic Hospital by Messrs. Gould and Sons.

